# Sensory and Motor Conduction Velocity in Spontaneously Hypertensive Rats: Sex and Aging Investigation

**DOI:** 10.3389/fnsys.2019.00062

**Published:** 2019-11-01

**Authors:** Lucas B. Fontanesi, Frederico S. Fazan, Fernando J. Dias, Maria Cristina L. Schiavoni, Wilson Marques Jr., Valéria Paula Sassoli Fazan

**Affiliations:** ^1^Department of Neuroscience and Behavioral Science, School of Medicine of Ribeirão Preto, University of São Paulo (USP), Ribeirão Preto, Brazil; ^2^Department of Physiology, Paulista School of Medicine, Federal University of São Paulo (UNIFESP), São Paulo, Brazil; ^3^Department of Integral Dentistry, CICO—Research Center in Dental Sciences, Dental School, Universidad de La Frontera (UFRo), Temuco, Chile; ^4^Department of Surgery and Anatomy, School of Medicine of Ribeirão Preto, University of São Paulo (USP), Ribeirão Preto, Brazil

**Keywords:** motor conduction velocity, sensory conduction velocity, SHR, aging, electrophysiology

## Abstract

The literature is extensive on how hypertension affects the morphology and function of the central nervous system (CNS) and is being focused on multiple organ damage involving the kidneys, heart, endothelium and retina. Hypertension damage to the peripheral nervous system is less explored in the literature. We have previously shown morphometric alterations in large and small caliber myelinated fibers of nerves in the adult spontaneously hypertensive rat (SHR). However, the functional correlation of these findings has not been explored. We performed an electrophysiological investigation of hind limb nerves in SHR of both genders in different ages. Normotensive Wistar-Kyoto (WKY) rats were used as controls. Electrophysiological recordings and determination of motor (MCV) and sensory (SCV) nerve conduction velocity were performed in the same animals at four different ages: 5, 8, 20 and 40 weeks after birth. Comparisons were made between ages, genders and animal strain. We showed a continuous body weight increase in adult life in all animals studied. MCV got stable at 20-week old hypertensive animals and continued to increase in normotensive ones. The SCV was constant between the ages of 20 and 40 weeks old in female SHR and decreased in male SHR while it continued to increase in WKY animals. The electrophysiological investigation of the nerves in WKY and SHR from both genders and different ages, associated with morphological and morphometric data from the literature suggest that hypertension affects the nerve function and might corroborate the development of a peripheral neuropathy.

## Introduction

As the adult population grows with increased life expectancy, cognitive impairment and dementia have become an increasingly common problem. Cerebrovascular disease has been accepted as one of the most common etiology of vascular cognitive impairment and dementia worldwide (Meissner, 2016) and it is known that midlife hypertension is a leading risk factor for late-life dementia (Santisteban and Iadecola, 2018). Hypertension is among the major risk factors for cerebrovascular disease and is a common cause of stroke and microanatomical and functional changes of cerebral circulation (Sabbatini et al., 1996), leaving the brain more prone to microaneurysms, microbleeds, ischemia, thrombosis, neuronal damage, lacunar infarcts and others (Tomassoni et al., 2004; Meissner, 2016).

The literature is extensive on how hypertension affects the morphology and function of the central nervous system (CNS) and is being focused on multiple organ damage involving the kidneys, heart, endothelium and retina (Cremer et al., 2016; Kuntz et al., 2018). An important feature of hypertension is the increase of vascular resistance despite the normal cardiac output. This increased resistance appears due to structural changes in the vascular system leading to the multiple organ damage in hypertension. Structural changes in the vascular system consist primary of increased thickness of the arterial wall and luminal narrowing (Sabbatini et al., 1996). In fact, vascular structural changes in the peripheral nervous system were previously described (Sabbatini et al., 1996; Sanada et al., 2012), similar to those of large vessels for target organs in hypertension. Thus, it can be expected that, as the CNS and other known target organs, the peripheral nervous system may also be sensitive to hypertension.

Previous reports suggested that hypertension could cause neuropathy in human beings (Chaco and Viskoper, 1971; Viskoper et al., 1971) but very little investigation on this issue was performed later. Laboratory animal models (Sabbatini et al., 1996; Fazan et al., 1999, 2001; Tomassoni et al., 2004; Fazan et al., 2005; Licursi de Alcântara et al., 2008; Rodrigues et al., 2011; Sanada et al., 2012; Nukada et al., 2016; da Silva et al., 2016) were investigated for the possibility of nerve fiber lesions in hypertension. For all these animal studies, the spontaneously hypertensive rats (SHR) were used for being considered a good model of human essential hypertension (Trippodo and Frohlich, 1981). Also, despite a large number of rodent models of hypertension (Lerman et al., 2019), the SHR is still the most common rat model used for investigation of hypertensive target-organ damage and its treatment (Nukada et al., 2016; Lerman et al., 2019). Despite the morphological descriptions of a mild neuropathy, with mainly thin myelinated fibers alterations, in several functional distinct peripheral nerves (sensory, motor and autonomic), information on functional nerve alterations, particularly involving nerve conduction velocity in the SHR is still scarce.

Another issue that deserves attention is the sex influence on blood pressure levels. Sex differences are an important feature of hypertension and cardiovascular diseases in humans (Lerman et al., 2019) and similar differences have been described in most animals, including rodents (Lerman et al., 2019) with most mechanisms responsible for the sex differences in hypertension being reported in rodent models (Lerman et al., 2019). Sex also influences in an extensive manner the peripheral nervous system alterations, and the way nerves recover after an injury. Several literature reports showed differences between sex in the re-myelination, pain sensitization, neural regulation of the vascular function, *in vitro* axonal growth, and conduction velocity of nerves (Kovacic et al., 2003; Blacklock et al., 2005; Li et al., 2006). Sex differences in nerve morphology of hypertensive animals have been described as related to sex differences in arterial pressure levels (Licursi de Alcântara et al., 2008; Rodrigues et al., 2011; Sanada et al., 2012) however, the influence of hypertension in nerve function of mammals is not totally investigated.

Thus, we aimed to investigate SHR nerve function by means of electrophysiological parameters of hind limb nerves. This is a longitudinal study that evaluated nerves of SHR over a time range including the pre-symptomatic period up to adulthood and aging. Comparisons with age-matched normotensive Wistar-Kyoto rats (WKY) were performed and the possible sex differences between animal strains were investigated.

## Materials and Methods

### Animals

SHR and WKY animals (sourced from the CEMIB—Multidisciplinary Center for Biological Research, State University of São Paulo—UNICAMP) were born and raised in a carefully controlled environment of light (12-h light/dark cycle), temperature (21–23°C), and relative air humidity (40%–70%), and received water and rat chaw (CR1 Nuvilab—Nuvital^®^) *ad libitum* throughout the experiment. Mature WKY females cohabitated with mature WKY males, derived from different mothers, for 21 days, for breeding purposes. For SHR, breeding was made between siblings as described (Okamoto and Aoki, 1963). Pregnant females were followed closely and after birth they were single-housed until their litter was weaned on the 30th day. After weaning, males and females of each strain were separated and housed in plastic cages (3–4 animals in each cage) under controlled conditions as described above. The litters were followed over time, from birth up to 40 weeks of life. The experiment started with a total of 89 rats, 44 males and 45 females, separated into four experimental groups as follows: female WKY (*n* = 16), female SHR (*n* = 29), male WKY (*n* = 15) and male SHR (*n* = 29). Due to initial difficulties in the obtention of an appropriate nerve recording in very young animals, the number of animals in the first group investigated was reduced as shown in Table 1. Further over time reductions in the number of animals occurred due to complications of needle insertion such as local hematoma formation and local infection, or natural death. Table 1 shows the number of animals per group used in each experimental time.

**Table 1:** Number of animals used in each experimental time.

	Female WKY	Male WKY	Female SHR	Male SHR
5 weeks	14	15	25	15
8 weeks	13	14	21	13
20 weeks	13	14	21	10
40 weeks	8	8	19	8

All experiments were approved by the Institutional Ethics Committee for Animal Research (CETEA–Comitê de Ética em Experimentacão Animal, protocol number 207/2009) from the School of Medicine of Ribeirão Preto, University of São Paulo, and were carried out according to the National Institutes of Health (NIH) Guide for the Care and Use of Laboratory Animals. All efforts were made to minimize the number of animals used.

### Electrophysiological Recordings

On the day before the electrophysiological studies, blood pressure was measured using the indirect method of tail-cuff plethysmography (B60-1/4, IITC Life Sciences Instruments, USA) with an IITC Life Sciences Model I-229 amplifier with automatic cuff inflation. The software used was an IITC Life Sciences Blood Pressure Data Acquisition Software. Since mean arterial pressure (MAP) in the plethysmography method strongly correlates with the gold standard direct (invasive) blood pressure measurement through arterial cannulation (Reddy et al., 2003; Kurtz et al., 2005) MAP data will be presented and discussed. Three consecutive measures of the MAP were obtained (three different instances of device-output MAP) and the average of them was used as the arterial pressure (MAP) presented in the results.

**Figure 1:**
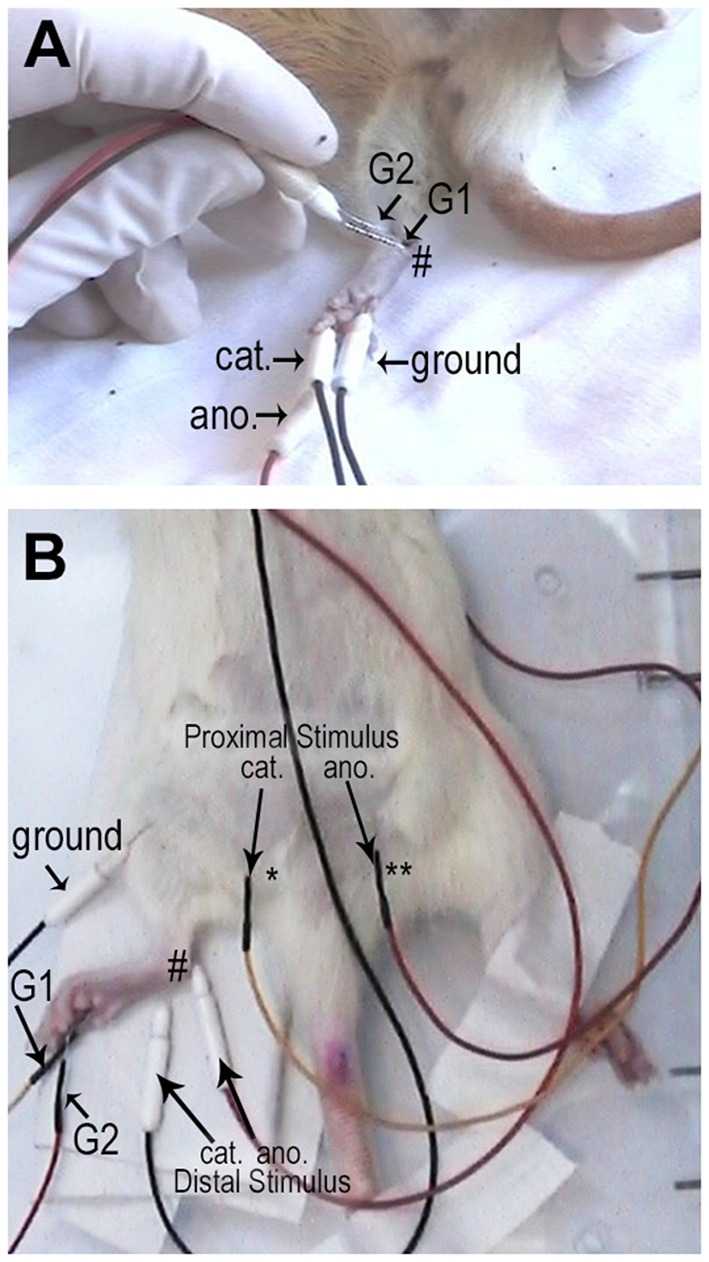
Representative pictures of the electrodes positioning for the sensory **(A)** and motor **(B)** conduction velocity recordings. For sensory conduction velocity (SCV), the active electrode (recording electrode—G1) was placed at the ankle (#; medial malleolus), the reference electrode (G2) was placed 1 cm far proximal, the cathode (cat) was inserted in the 5th toe and the anode (ano.) in the 3rd toe. A cooper ground electrode (grd.) was inserted subcutaneously in the posterior side of the leg, opposing the recording electrode. For motor conduction velocity (MCV) the active recording electrode (G1) was positioned in the plantar muscles and the reference electrode (G2) was positioned on the 4th toe. For the distal stimulation, the cathode (cat.) was positioned at the ankle (#) while the anode (ano.) was positioned 1 cm proximally; while for proximal stimulation, the cathode (cat.) was positioned at the sciatic notch (*) and the anode was positioned contra-laterally ** (ano.).

For electrophysiological recordings and determination of motor (MCV) and sensory (SCV) nerve conduction velocity a Keypoint 4, Dantec-Medtronic^®^ electroneuromyography device was used. The exams were performed in the same animals in four different ages: 5, 8, 20 and 40 weeks after birth. Hind limb skin temperature was kept above 33°C throughout the recordings with a heating pad. Animals were anesthetized with Ketamine 5% and Xylazine 2% (1:4) i.p. (0.1 ml/100 g of body weight) and subdermal needle electrodes (used both to stimulate and to record) were positioned as follows (Figure 1). Methods for measuring SCV and MCV were adapted from those previously described in the literature for rats (Baba et al., 1982, 2006; Nukada et al., 2016).

For SCV, the active electrode (recording electrode) was placed at the ankle (medial malleolus), the reference electrode was placed 1 cm far proximal, the cathode was inserted in the 5th toe and the anode in the 3rd toe. A cooper ground electrode (connected to six cooper rods) was inserted subcutaneously in the posterior side of the leg, opposing the recording electrode. The latency (ms) was measured from the stimulation artifact to the first positive spike. The amplitude (mV) was measured from the first positive spike to the negative spike. SCV (m/s) was calculated by dividing the distance between the recording electrodes by the latency of the signal from the stimulation artifact to the onset of the sensory nerve action potential. Measurements were made with a caliper compass and the hint was kept always at the same position. Electric stimuli were always supramaximal. Stimuli duration was with 0.05 or 0.1 ms duration, filters were kept between 20 Hz to 5 kHz. Gain was set to 20 μV/division and sweep velocity was set to 1 ms/division.

For MCV, the active recording electrode was positioned in the plantar muscles and the reference electrode was positioned on the 4th toe. For the distal stimulation, the cathode was positioned at the ankle while the anode was positioned 1 cm proximally; while for proximal stimulation the cathode was positioned at the sciatic notch and the cathode was positioned contra-laterally. Electric stimuli were always supramaximal (determined for each animal; 0.2–0.6 mA) with 0.05 or 0.1 ms duration. Care was taken to avoid muscle artifacts. Filters were kept between 10 Hz to 10 KHz. Gain was set to 2 mV/division and sweep velocity was set to 1 ms/division. The active electrode positioning was optimized in order to obtain a compound muscle action potential with negative initial deflexion. The latency (ms) was measured from the stimulation artifact to the initial negative deflexion. The amplitude (mV) was measured from the baseline to the negative spike. MCV (m/s) was calculated dividing the distance between the distal and the proximal cathodes by the difference between proximal and distal latencies. For distance measurements, hip was kept ant 90° and the hint was straightened until a pressure was felt.

### Data Analysis

Data were tested for normal distribution by the Kolmogorov-Smirnov normality test followed by the Levene test for variance equivalence using the Sigma-Stat (v.3.5, Jandel Scientific) software. Comparisons were made between sexes in the same strain and between different strains in animals with same sex and same age by unpaired Student’s *t*-test if data presented a normal distribution and equivalent variance. Otherwise, comparisons were made by the Mann–Whitney non-parametric test. For comparisons between ages in the same strain and sex, a one-way analysis of variance (ANOVA) for repeated measures followed by the Holm–Sidak post-test was used.

The relationship between body weight and sensory (SCV) or motor (MCV) conduction velocity was investigated by plotting the data on scatter diagrams (y = body weight and x = conduction velocity), using the Sigma Plot^®^ 13 [Systat Software Inc. (SSI), San Jose, CA, USA]. The same investigation was performed for the relationship between MAP and SCV or MCV (y = mean arterial pressure and x = conduction velocity). A regression line was fitted by the least-squares method for the plotted data for each experimental group as described (Fazan et al., 2005; Sato et al., 2018). All linear regression lines were tested for normality using the Shapiro–Wilk test. A constant variance test was also applied to each regression. The Pearson product-moment correlation (*r*, to measure the degree of association between the two investigated variables) was calculated for each scatter diagram (Sato et al., 2018).

Differences were considered significant if *p* < 0.05. Data are presented as mean ± standard error of the mean (SEM).

## Results

**Figure 2:**
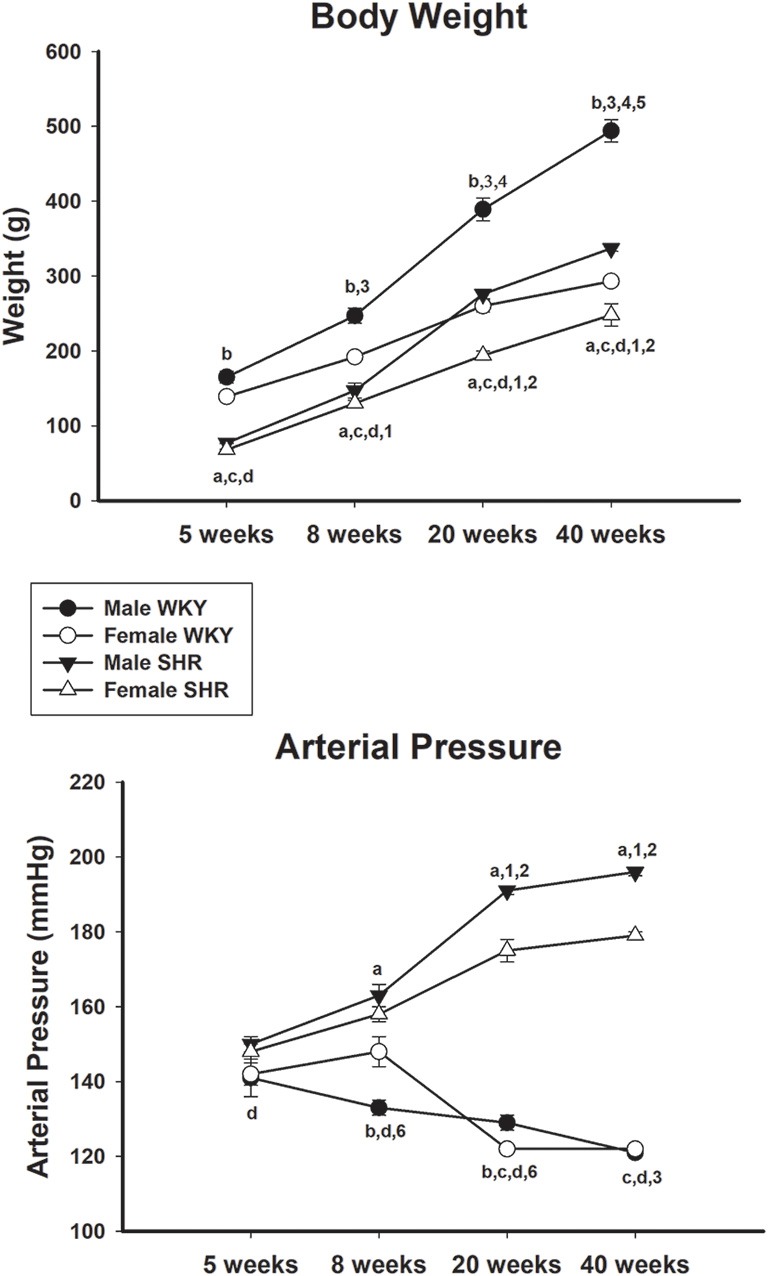
Average body weight (upper panel) and mean arterial pressure (MAP; lower panel) of male and female Wistar-Kyoto (WKY) and spontaneously hypertensive rats (SHR) at ages 5, 8, 20 and 40 weeks. a = *P* < 0.05 between male and female SHR (unpaired Student’s *t*-test); b = *P* < 0.05 between male and female WKY (unpaired Student’s *t*-test); c = *P* < 0.05 between females, same age (unpaired Student’s *t*-test); d = *P* < 0.05 between males, same age (unpaired Student’s *t*-test); 1 = *P* < 0.05 compared to 5 weeks old SHR [one-way analysis of variance (ANOVA), followed by Holm–Sidak post-test]; 2 = *P* < 0.05 compared to 8 weeks old SHR (one-way ANOVA, followed by Holm–Sidak post-test); 3 = *P* < 0.05 compared to 5 weeks old WKY (one-way ANOVA, followed by Holm–Sidak post-test); 4 = *P* < 0.05 compared to 8 weeks old WKY (one-way ANOVA, followed by Holm–Sidak post-test); 5 = *P* < 0.05 compared to 20 weeks old WKY (one-way ANOVA, followed by Holm–Sidak post-test); 6 = *P* < 0.05 compared to 5 weeks old WKY only in females (one-way ANOVA, followed by Holm–Sidak post-test). Data presented as mean ± standard error of the mean (SEM).

Body weight increased continuously in male and female animals from both strains throughout the experiment (Figure 2, upper panel) with statistical significance between all compared ages. There was a difference between genders when compared with the same strain in all ages. The comparison between the same gender and different strains showed that male WKY was notably heavier than male SHR in all ages while for females, the same difference was observed except for the age of 40 weeks.

The blood pressure data of male and female SHR and WKY from different ages are shown in Figure 2 (lower panel). Male and female WKY animals showed MAP values of 141 ± 5 mmHg and 142 ± 3, respectively at 5 weeks of age. MAP values decreased continuously with age, reaching statistical significance when compared ages 5 and 40 weeks, for both genders (one-way ANOVA, *p* < 0.001 for both, followed by Holm–Sidak post-test, *p* = 0.017 for females and *p* = 0.009 for males). Interestingly, females WKY had a significant increase in the MAP at 8 weeks of age, compared to males of the same strain, reaching similar values to males at ages 20 and 40 weeks (Figure 2, lower panel). For SHR, MAP was generally higher than WKY animals in all ages but became remarkably higher in both genders at ages 20 and 40 weeks, compared to WKY. Also, differences were observed between SHR genders, being MAP values from males significantly higher than females at ages 8, 20 and 40 weeks.

**Figure 3:**
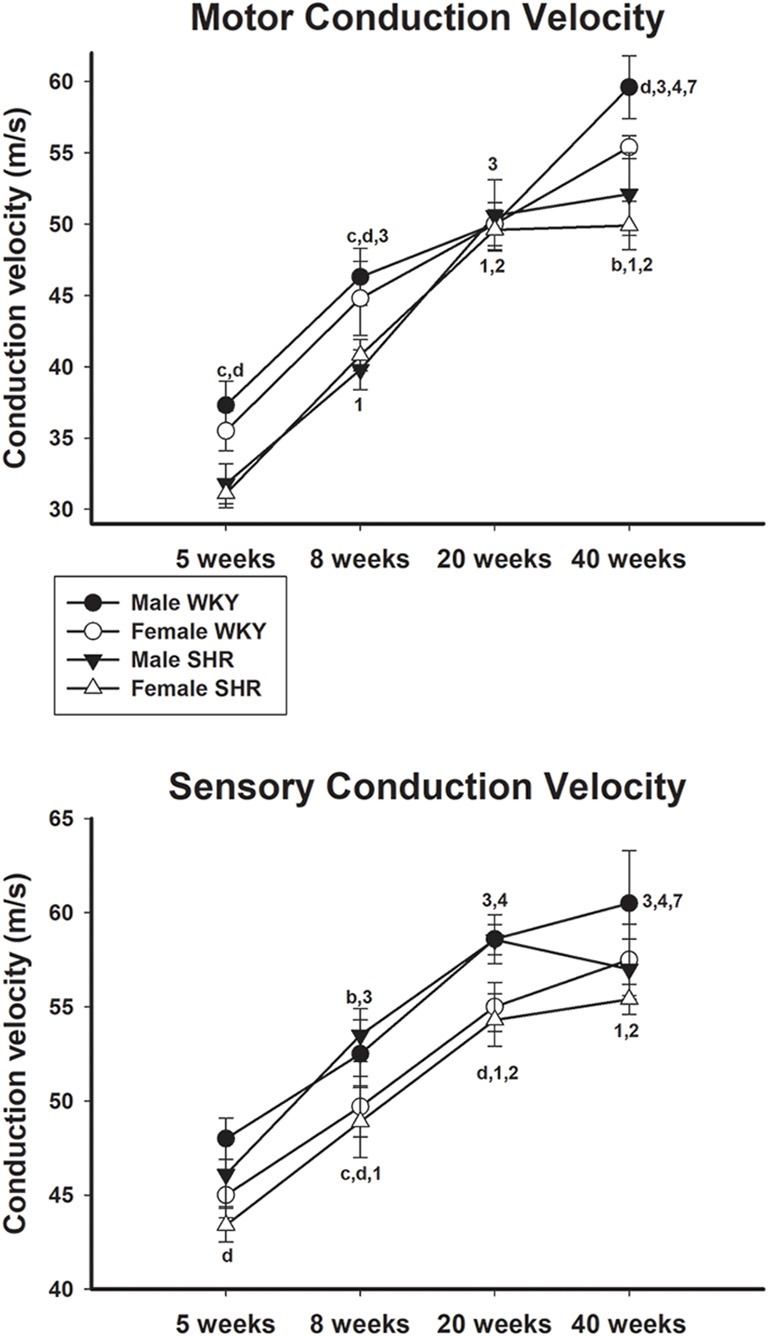
Average motor (upper panel) and sensory (lower panel) nerve conduction velocity of male and female WKY and SHR at ages 5, 8, 20 and 40 weeks. b = *P* < 0.05 between male and female WKY (unpaired Student’s *t*-test); c = *P* < 0.05 between females, same age (unpaired Student’s *t*-test); d = *P* < 0.05 between males, same age (unpaired Student’s *t*-test); 1 = *P* < 0.05 compared to 5 weeks old SHR (one-way ANOVA, followed by Holm–Sidak post-test); 2 = *P* < 0.05 compared to 8 weeks old SHR (one-way ANOVA, followed by Holm–Sidak post-test); 3 = *P* < 0.05 compared to 5 weeks old WKY (one-way ANOVA, followed by Holm–Sidak post-test; one-way ANOVA, followed by Holm–Sidak post-test); 4 = *P* < 0.05 compared to 8 weeks old WKY (one-way ANOVA, followed by Holm–Sidak post-test); 7 = *P* < 0.05 compared to 20 weeks old WKY only in males (one-way ANOVA, followed by Holm–Sidak post-test). Data presented as mean ± SEM.

Motor and sensory conduction velocity data of male and female SHR and WKY from different ages are presented in Figure 3, upper and lower panels, respectively. Motor conduction velocity increased with aging in both genders either in SHR or WKY strains. For SHR animals, differences in the MCV were not present between 20- and 40-week-old animals in both genders. For the SCV in SHR, males showed an increase only between ages 5 and 8 weeks. In general, values were similar between genders in the same strain but higher in WKY compared to SHR in both genders. For WKY animals, SCV also increased with age, with male values being generally higher than females. Nevertheless, for male SHR this velocity showed an abrupt decrease from 20 to 40 weeks of age, which was not observed in female SHR (no difference between 20 and 40 weeks) or WKY animals.

**Figure 4:**
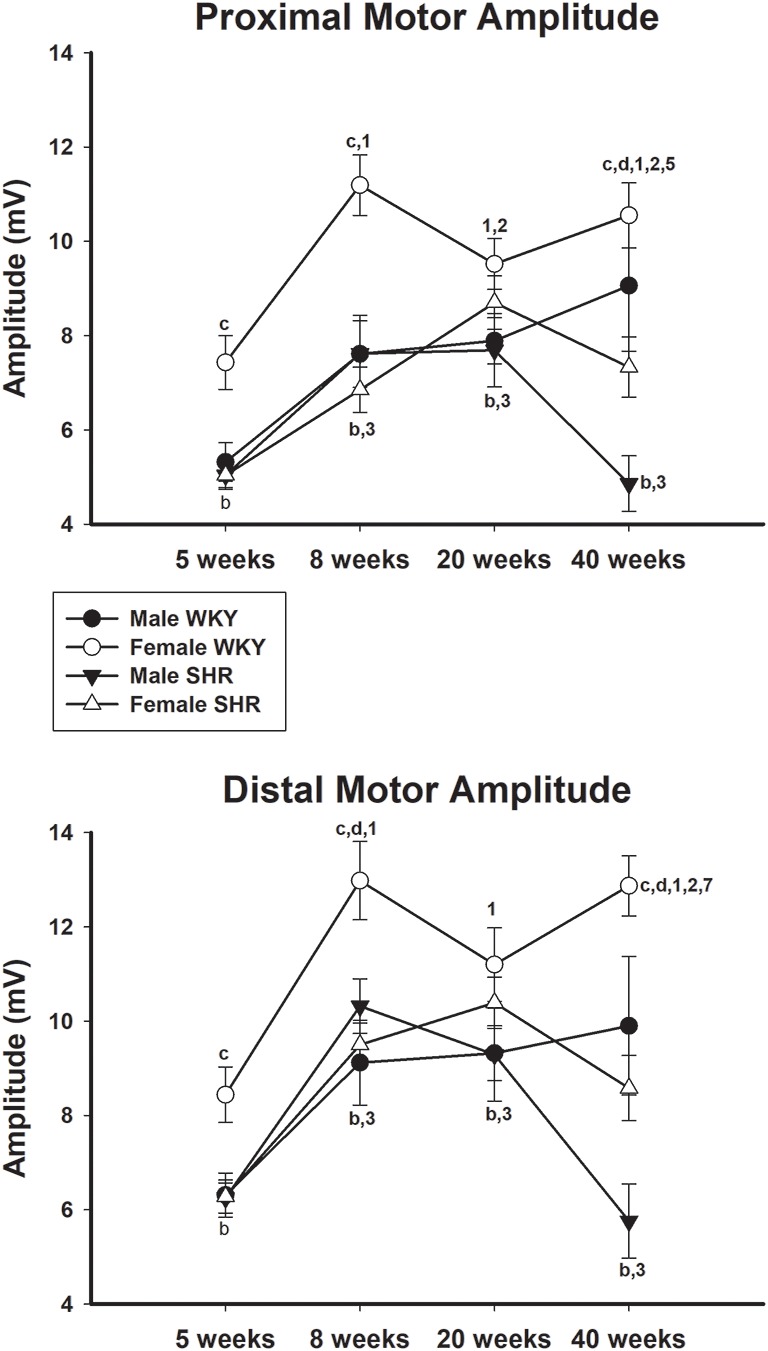
Average proximal (upper panel) and distal (lower panel) motor amplitude in male and female WKY and SHR at ages 5, 8, 20 and 40 weeks. b = *P* < 0.05 between male and female WKY (unpaired Student’s *t*-test); c = *P* < 0.05 between females, same age (unpaired Student’s *t*-test); d = *P* < 0.05 between males, same age (Mann-Whitney non-parametric test); 1 = *P* < 0.05 compared to 5 weeks old SHR (one-way ANOVA, followed by Holm–Sidak post-test); 2 = *P* < 0.05 compared to 8 weeks old SHR (one-way ANOVA, followed by Holm–Sidak post-test); 3 = *P* < 0.05 compared to 5 weeks old WKY (one-way ANOVA, followed by Holm–Sidak post-test); 4 = *P* < 0.05 compared to 8 weeks old WKY (one-way ANOVA, followed by Holm–Sidak post-test); 7 = *P* < 0.05 compared to 20 weeks old WKY only in males (one-way ANOVA, followed by Holm–Sidak post-test). Data presented as mean ± SEM.

**Figure 5:**
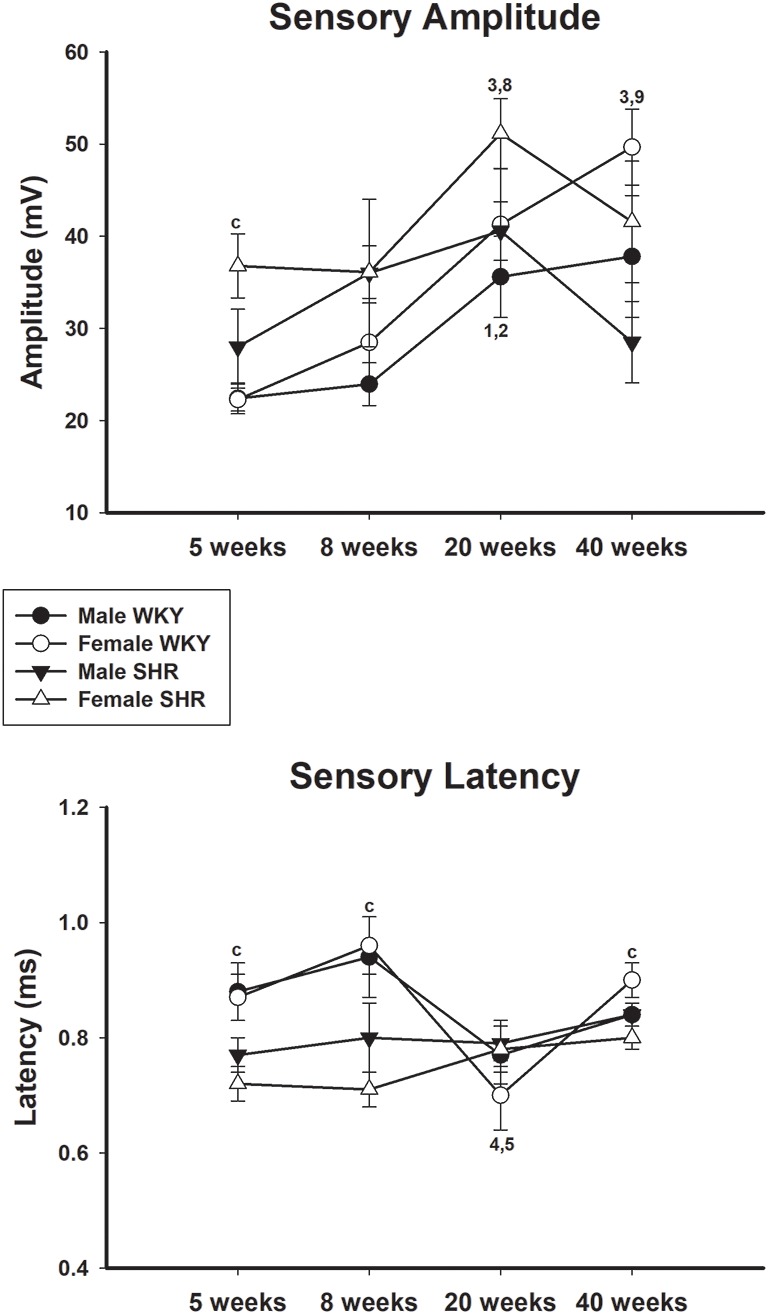
Average sensory amplitude (upper panel) and sensory latency (lower panel) in male and female WKY and SHR at ages 5, 8, 20 and 40 weeks. c = *P* < 0.05 between females, same age (unpaired Student’s *t*-test); 1 = *P* < 0.05 compared to 5 weeks old SHR (one-way ANOVA, followed by Holm–Sidak post-test); 2 = *P* < 0.05 compared to 8 weeks old SHR (one-way ANOVA, followed by Holm–Sidak post-test); 3 = *P* < 0.05 compared to 5 weeks old WKY (one-way ANOVA, followed by Holm–Sidak post-test); 4 = *P* < 0.05 compared to 8 weeks old WKY (one-way ANOVA, followed by Holm–Sidak post-test); 5 = *P* < 0.05 compared to 20 weeks old WKY (one-way ANOVA, followed by Holm–Sidak post-test); 8 = *P* < 0.05 compared to 8 weeks old WKY only in males (one-way ANOVA, followed by Holm–Sidak post-test); 9 = *P* < 0.05 compared to 8 weeks old WKY only in females (one-way ANOVA, followed by Holm–Sidak post-test). Data presented as mean ± SEM.

Average values of proximal and distal motor amplitudes of male and female SHR and WKY from different ages are presented in Figure 4, upper and lower panels, respectively. Female WKY showed generally higher values for either proximal or distal motor amplitudes, compared to other groups. For WKY animals, values increased with age while for SHR animals there was a decrease of values from 20 to 40 weeks of age, for both genders, but more pronounced in males. Sensory amplitude average values (Figure 5, upper panel) followed the same trend as motor amplitude, showing increased values with aging for WKY and a decrease from 20 to 40 weeks of age in SHR animals.

**Figure 6:**
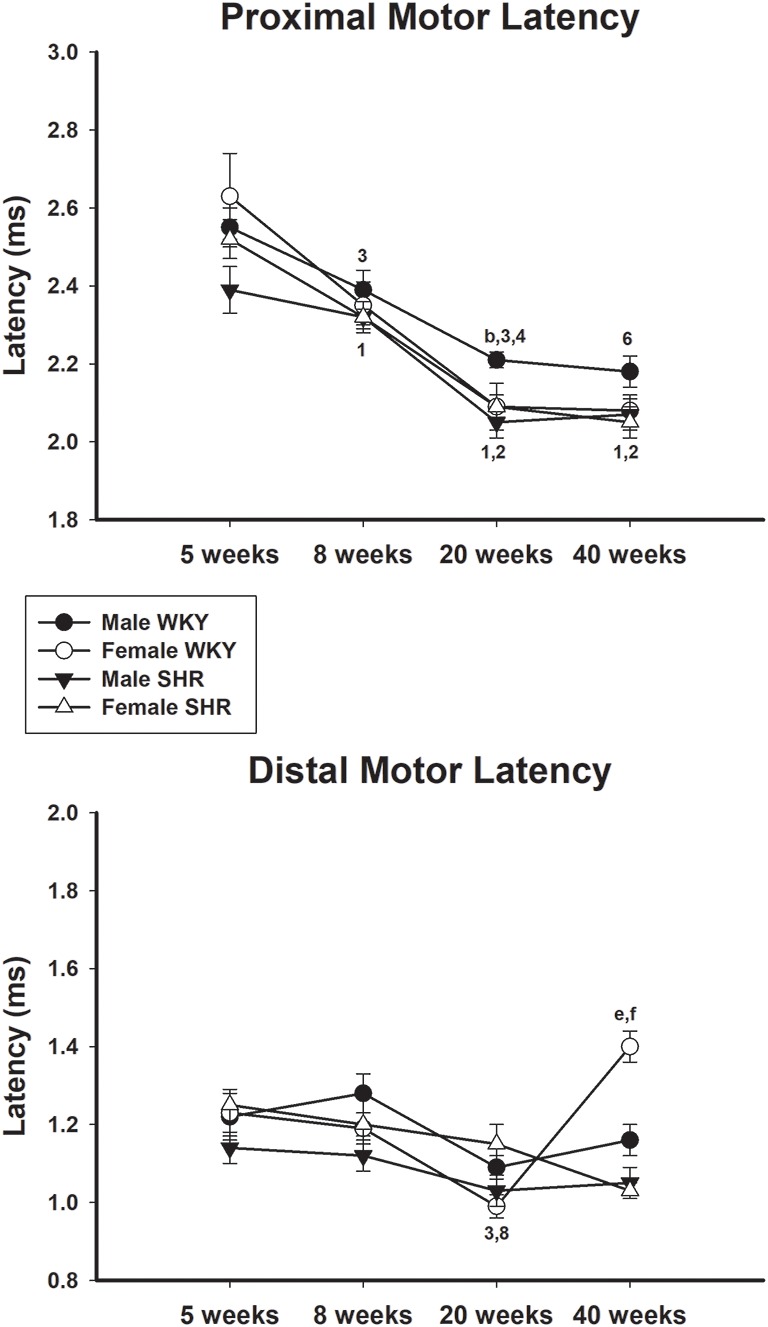
Average proximal (upper panel) and distal (lower panel) motor latency in male and female WKY and SHR at ages 5, 8, 20 and 40 weeks. b = *P* < 0.05 between male and female WKY (Mann-Whitney non-parametric test); 1 = *P* < 0.05 compared to 5 weeks old SHR (one-way ANOVA, followed by Holm–Sidak post-test); e = *P* < 0.05 compared to 5 weeks old SHR only in females (one-way ANOVA, followed by Holm–Sidak post-test); 2 = *P* < 0.05 compared to 8 weeks old SHR (one-way ANOVA, followed by Holm–Sidak post-test); f = *P* < 0.05 compared to 8 weeks old SHR only in females (one-way ANOVA, followed by Holm–Sidak post-test); 3 = *P* < 0.05 compared to 5 weeks old WKY (one-way ANOVA, followed by Holm–Sidak post-test); 4 = *P* < 0.05 compared to 8 weeks old WKY (one-way ANOVA, followed by Holm–Sidak post-test); 6 = *P* < 0.05 compared to 5 weeks old WKY only in females (one-way ANOVA, followed by Holm–Sidak post-test); 8 = *P* < 0.05 compared to 8 weeks old WKY only in females (one-way ANOVA, followed by Holm–Sidak post-test). Data presented as mean ± SEM.

Average values of proximal and distal motor latency of male and female SHR and WKY from different ages are presented in Figure 6, upper and lower panels, respectively. Proximal motor latency values decreased with age with this decrease being less evident in male WKY at 20 and 40 weeks of age. Distal motor latency values also decreased with age but varied less between age in all groups, reaching higher values in female WKY at 40 weeks of age compared to female SHR. Sensory latency average values (Figure 5, lower panel) showed higher values in WKY animals particularly in females compared to SHR at ages 5 and 8 weeks and reached similar values at older ages.

The relationship between body weight and sensory (SCV) or motor (MCV) conduction velocity, presented as the Pearson product-moment correlation (*r*-value) is shown in Table 2. With few exceptions (indicated in Table 2), all linear regression lines results passed the Shapiro–Wilk test. Values ranged from 0.05 to 0.625 for MCV and from 0.013 to 0.634, with no trends between sex, age or animal strain.

**Table 2:** Correlation coefficient (*r*-values) for linear regression between body weight and sensory (SCV) or motor (MCV) conduction velocities in each experimental time.

	Female WKY	Male WKY	Female SHR	Male SHR
	MCV/SCV	MCV/SCV	MCV/SCV	MCV/SCV
5 weeks	0.706/0.230	0.611/0.313*	0.050/0.013	0.194/0.257
8 weeks	0.127/0.364	0.566*/0.408	0.275*/0.232*	0.189/0.318
20 weeks	0.144/0.342	0.665/0.513	0.534/0.105*	0.304/0.245
40 weeks	0.550/0.226	0.475/0.110	0.625/0.144*	0.602/0.123

*Indicates failure in the Shapiro Wilk normality test.

The relationship between MAP and sensory (SCV) or motor (MCV) conduction velocity, presented as the Pearson product-moment correlation (*r*-value) is shown in Table 3. With few exceptions (indicated in Table 3), all linear regression lines results passed the Shapiro–Wilk test. The correlation values indicated a week to moderate relation between variables (most values between 0.5 and 0.7) with a possible relation between MCV and the MAP for the WKY animals from ages 8 and 20 weeks. No relation could be detected for SRH nerves in all ages from both sexes. Nevertheless, a blunt decrease in the *r*-value could be observed in 20-week-old animals, compared to the other ages, for the MCV.

**Table 3:** Correlation coefficient (*r*-values) for linear regression between mean arterial pressure (MAP) and sensory (SCV) or motor (MCV) conduction velocities in each experimental time.

	Female WKY	Male WKY	Female SHR	Male SHR
	MCV/SCV	MCV/SCV	MCV/SCV	MCV/SCV
5 weeks	0.337/0.263*	0.467/0.459	0.0265/0.0168	0.222*/0.131
8 weeks	0.676/0.238*	0.654/0.379	0.109/0.214	0.488*/0.295
20 weeks	0.623/0.563	0.648/0.176	0.167/0.253	0.088/0.362
40 weeks	0.397*/0.250	0.365/0.349	0.410/0.310	0.388/0.333

*Indicates failure in the Shapiro Wilk normality test.

## Discussion

This study shows functional alterations of nerves from hypertensive animals compatible with previous morphologic and morphometric descriptions of a mild neuropathy in SHR (Fazan et al., 1999; Rodrigues et al., 2011; Sanada et al., 2012). Moreover, differences between males and females SHR were present at 40-week-old animals, with impaired nerve function more pronounced in males. This is in line with differences in blood pressure levels between male and female SHR, being higher in males after 8 weeks of age.

Our results showed differences in body weight between male and female SHR and WKY, being males havier than females in both strains. Similar findings were previously described in our laboratory (Licursi de Alcântara et al., 2008; Rodrigues et al., 2011; Sanada et al., 2012; da Silva et al., 2016) and others (Calhoun et al., 1995; Kauser and Rubanyi, 1995; Chen et al., 1997; Chandler and DiCarlo, 1998; Kähönen et al., 1998; Radin et al., 2002; Vlkovicová et al., 2005). Also, WKY animals from both sexes were heavier than SHR, compatible to previous literature reports (Calhoun et al., 1995; Kauser and Rubanyi, 1995; Chen et al., 1997; Chandler and DiCarlo, 1998; Kähönen et al., 1998; Bacáková and Kunes, 2000; Vlkovicová et al., 2005; Rodrigues et al., 2011; Sanada et al., 2012).

To ensure that body weight differences did not create a bias on the nerve conduction velocity study, the correlation between the two variables was investigated in all groups (Table 2). Despite that the correlation coefficients (*r*-value) were positive, all of them showed very low values, indicating a week relation between body weight and either sensory or motor conduction velocities. This is a possible indication that alterations described for functional parameters of the SHR nerves could be due to the hypertension development, instead of the animal size increase only.

Morphometric differences between males and females adult SHR were previously demonstrated in the vagus (Licursi de Alcântara et al., 2008), phrenic (Rodrigues et al., 2011) and sural nerves (Sanada et al., 2012). In 20 week-old SHR, total myelinated fiber number was smaller in the vagus and sural nerves of male SHR, reaching statistical difference in the phrenic nerves. Also, the number and density of the Schwann cell nuclei were significantly larger in male SHR vagus nerves, compared to females at older ages (Licursi de Alcântara et al., 2008), suggesting an alteration of, not only the nerve fibers but also the endoneural content due to hypertension. Thus, if the peripheral nerves of adult SHR show morphometric differences between sexes, we would expect to find functional differences between sexes as well. In fact, functional differences between SHR sexes were found and, more importantly, differences between hypertensive and normotensive animals were also present as discussed below.

It is well described that female rats are less susceptible to the development of spontaneous neuropathy (Majeed, 1992). Also, female rats live longer and in better condition than males (Van Steenis and Kroes, 1971). Peripheral nerve alterations due to aging were described for male (Sharma et al., 1980) and female rats (Jeronimo et al., 2005, 2008) and found to be different between motor (Sharma et al., 1980) and sensory (Jeronimo et al., 2005, 2008) nerves. Also, despite mild changes, nerves from males are more affected than those of females of the same age, with no differences between proximal and distal nerve segments (Van Steenis and Kroes, 1971). Nevertheless, those studies investigated animals with 1 year of age or older. That is not the case in this study since the older animals were 40 weeks of age. Because of the young age investigated in the present study, one would not expect to find nerve alterations due to aging. The most important variable that could introduce the observed alterations in our experimental protocol is the development of spontaneous hypertension and the sustained high blood pressure over time.

Conventional methods for measuring the motor conduction velocity tend to reflect primary on fast conduction fibers (Ikeda and Oka, 2012). Thus, increase in the motor conduction velocity values reflects the relative increase in the number of large diameter fibers detected in a nerve (Ikeda and Oka, 2012). An increase in nerve conduction velocity is expected during the young adult life of rats (between 2–6 months of age) suggesting a long period of postnatal maturation of nerve fibers that affect fiber size and function.

Increased motor conduction velocity shown in this study for older animals independently of strain or gender reflects normal morphometric alterations in nerves since there is an expected increase in myelinated fiber size with aging. Despite our results of a continuous body weight increase in adult life in all animals studied (Figure 2), motor conduction velocity got stable at 20-week old hypertensive animals but continued to increase in normotensive ones (Figure 3), becoming statistically higher in 40 weeks old WKY compared to SHR. This is in agreement with previous nerve morphology results of thinning of the large myelinated fibers observed at 6 months of age (Tomassoni et al., 2004) and myelin splitting observed at 44 weeks of age (Nukada et al., 2016) in SHR with well-established hypertension. It is important to point out that the MCV values obtained in our normotensive rats are very similar to those reported for normotensive Wistar rats or WKY rats with similar ages investigated in the present study. Baba et al. (2006) reported MCV values of 53.6 ± 3.4 m/s for 15-week-old Wistar rats and Nukada et al. (2016) reported a MCV of 43.4 ± 0.7 m/s and 63.5 ± 0.9 m/s for WKY rats aged 8 and 44 weeks, respectively. Our MCV average values (shown in Figure 3) for 8, 20 and 40 weeks old normotensive animals were 44.8 ± 2.6, 50.0 ± 1.5 and 55.5 ± 0.8 m/s, respectively.

It is known that the MCV remains unchanged during adulthood in normal rats, until the last third of the life, when it tends to decline (Verdú et al., 2000). In our study, the decline observed in SHR animals was much earlier (between 20 and 40 weeks of age) than that expected for normal rodents (after 20 months of life). The relation between the increase in body weight and increase motor conduction velocity was disrupted in 40 weeks old SHR, suggesting that after the stabilization of the high blood pressure (after 20 weeks of age), nerve fibers might show impaired function. The relation between MAP and MCV was positive for all groups. This was not expected and further investigation will be necessary to better understand this result. Nevertheless, while week to moderate correlations were present in normotensive rats, the correlations for SHR were very week, getting the lowest values in 20 weeks old male SHR. This might be suggestive of a disruption of this relation when MAP gets set to high values or can also indicate that this is the age when fibers present signs of functional damage. Another possibility that can not be excluded is the presence of intrinsic differences between stains. As one limitation of our study, we did not access fiber damage directly, because of the particular interest in following the increase in blood pressure and changes in the conduction velocity over time in the same animals.

In line with the results of motor conduction velocity, proximal and distal motor amplitudes were significantly reduced in SHR in both genders at 40 weeks of age, compared to WKY animals. Differences were less pronounced for latency data, despite the expected reduction of the values with age. Proximal and distal latencies decreased with age in all experimental groups and this decrease was slightly more pronounced in the proximal segment. Reduction of latency values in older animals is due to the myelinated fiber maturation since it is well known that myelinated fibers reduce their growth rate (percentage size of the total fiber size; Schäfer and Friede, 1988). with aging (Sharma et al., 1980; Jeronimo et al., 2005, 2008). It has been demonstrated that myelinated fibers grow rapidly between ages of 30 and 90 days (similar to ages 5–8 weeks from the present study; Jeronimo et al., 2005) and get at a stable size at 180 days (about 20 weeks of age from the present study; Sharma et al., 1980; Jeronimo et al., 2008), but continue to grow very slowly up to 1 year of age (over 40 weeks of age compared to the present study).

Sensory conduction velocity also increased with age (Figure 3), but younger animals (5 and 8 weeks old) started at higher values when compared to the motor conduction velocity and also got stable at lower values in all experimental groups. This is expected since sensory fibers’ size increase with aging but stops growing at lower values than motor fibers (Schäfer and Friede, 1988). This observation on sensory conduction velocity being lower than motor conduction velocity at older ages was also described for normotensive Wistar rats (Sato et al., 1985) and for healthy asymptomatic humans (Stetson et al., 1992). Early-life differences between SHR and WKY were observed in a similar way to those of the motor conduction velocity. Nevertheless, between the ages of 20 and 40 weeks old, female SHR showed a constant sensory conduction velocity while males presented a significant reduction on this parameter. It continued to increase as expected in WKY animals. This result is absolutely in line with the morphometric observation on sural nerves of adult SHR, which showed reduction of fiber number, particularly the small myelinated ones (Sanada et al., 2012). Those authors also demonstrated that sural nerves of adult SHR showed larger average fiber diameter in SHR (Sanada et al., 2012), pointing to a reduction of the small fibers. Associated with the fiber number reduction, a larger average myelinated fiber size is indicative of small fiber loss with hypertension. This is in line with the reduction of the sensory conduction velocity in SHR shown in this study. Sensory amplitude values increase with aging but also reached smaller values in SHR compared to WKY at 40 weeks of age. Ayaz et al. (2007) described that the necessary time to reach maximal depolarization of nerve fibers in females is larger than males in 12-week-old normotensive rats. This finding is also a normal characteristic of the sural nerve in healthy humans (Stetson et al., 1992). This is similar to the WKY results but not to SHR. Statistic difference in sensitive latency was present in females, being larger in WKY than SHR. Once again, this is in line with slight gender differences in sural nerve morphology in SHR (Sanada et al., 2012).

In conclusion, the electrophysiological investigation of the nerves in WKY and SHR from both genders and different ages, associated with morphological and morphometric data from the literature suggest that hypertension affects the nerve function and might corroborate for a neuropathy development.

## Data Availability Statement

The datasets generated for this study are available on request to the corresponding author.

## Ethics Statement

The animal study was reviewed and approved by CETEA–Comitê de Ética em Experimentacão Animal, protocol number 207/2009, from the School of Medicine of Ribeirão Preto, University of São Paulo.

## Author Contributions

LF and FF: responsible for the acquisition of data, analysis, and interpretation. FD and WM: responsible for the acquisition of data and analysis. MS: responsible for the acquisition of data. VF: responsible for conception and design, interpretation of data, drafting the manuscript and for the final approval of the version to be published.

## Conflict of Interest

The authors declare that the research was conducted in the absence of any commercial or financial relationships that could be construed as a potential conflict of interest.
